# Alterations in mitochondrial respiration and reactive oxygen species in patients poisoned with carbon monoxide treated with hyperbaric oxygen

**DOI:** 10.1186/s40635-018-0169-2

**Published:** 2018-01-30

**Authors:** David H. Jang, Utsha G. Khatri, Brenna P. Shortal, Matthew Kelly, Kevin Hardy, David S. Lambert, David M. Eckmann

**Affiliations:** 1 Department of Emergency Medicine, Division of Medical Toxicology and Critical Care Medicine, Perelman School of Medicine, University of Pennsylvania, John Morgan Building, 3620 Hamilton Walk, Philadelphia, 19104 PA USA; 2 Department of Anesthesiology and Critical Care, Perelman School of Medicine, University of Pennsylvania, Philadelphia, 19104 PA USA; 3 Department of Bioengineering, School of Engineering and Applied Sciences, University of Pennsylvania, Philadelphia, 19104 PA USA; 4 Department of Emergency Medicine, Division of Hyperbaric and Undersea Medicine, Perelman School of Medicine, University of Pennsylvania, Philadelphia, USA; 5 Institute for Medicine and Engineering, University of Pennsylvania, Philadelphia, USA; 6 Institute for Translational Medicine and Therapeutics, University of Pennsylvania, Philadelphia, USA; 7 Cardiovascular Institute, University of Pennsylvania, Philadelphia, USA

**Keywords:** Mitochondria, Carbon monoxide, Reactive oxygen species, Hyperbaric oxygen

## Abstract

**Background:**

Carbon monoxide (CO) poisoning is the leading cause of poisoning mortality and morbidity in the USA. Carboxyhemoglobin (COHb) levels are not predictive of severity or prognosis. At this time, the measurement of mitochondrial respiration may serve as a biomarker in CO poisoning. The primary objective of this study was to assess changes in mitochondrial function consisting of respiration and generation of reactive oxygen species (ROS) in peripheral blood mononuclear cells (PBMCs) obtained from patients with CO poisoning.

**Methods:**

PBMCs from patients having confirmed CO exposure treated with hyperbaric oxygen or HBO (CO group) and healthy controls (control group) were analyzed with high-resolution respirometry. PBMCs were placed in a 2-ml chamber at a final concentration of 3–4 × 10^6^ cells/ml to simultaneously obtain both respiration and hydrogen peroxide (H_2_O_2_) production. In the CO group, we performed measurements before and after patients underwent their first HBO treatment.

**Results:**

We enrolled a total of 17 subjects, including 7 subjects with confirmed CO poisoning and 10 subjects in the control group. The CO group included five (71.4%) men and two (28.6%) women having a median COHb of 28%. There was a significant decrease in respiration as measured in pmol O_2_ × s^− 1^ × 10^− 6^ PBMCs in the CO group (pre-HBO) when compared to the control group: maximal respiration (18.4 ± 2.4 versus 35.4 ± 2.8, *P* < 0.001); uncoupled Complex I respiration (19.8 ± 1.8 versus 41.1 ± 3.8, *P <* 0.001); uncoupled Complex I + II respiration (32.3 ± 3.2 versus 58.3 ± 3.1, *P <* 0.001); Complex IV respiration (43.5 ± 2.9 versus 63.6 ± 6.31, *P* < 0.05). There were also similar differences measured in the CO group before and after HBO treatment with an overall increase in respiration present after treatment. We also determined the rate of H_2_O_2_ production simultaneously with the measurement of respiration. There was an overall significant increase in the H_2_O_2_ production in the CO group after HBO treatment when compared to prior HBO treatment and the control group.

**Conclusions:**

In this study, PBMCs obtained from subjects with CO poisoning have an overall decrease in respiration (similar H_2_O_2_ production) when compared to controls. The inhibition of Complex IV respiration is from CO binding leading to a downstream decrease in respiration at other complexes. PBMCs obtained from CO-poisoned individuals immediately following initial HBO therapy displayed an overall increase in both respiration and H_2_O_2_ production. The study findings demonstrate that treatment with HBO resulted in improved cellular respiration but a higher H_2_O_2_ production. It is unclear if the increased production of H_2_O_2_ in HBO treatment is detrimental.

## Background

Carbon monoxide (CO) is a colorless and odorless gas that is an important cause of poisoning mortality and morbidity in the USA with approximately 15,000 intentional cases annually accounting for over two thirds of reported death. Specifically, death from CO poisoning has been reported to be between 1000 to 2000 in some years with over 50,000 CO cases seen in emergency departments in the USA annually, with approximately 10–15% requiring hospitalization [1, 2]. It is estimated that CO poisoning results in over $1 billion annually related to hospital costs and lost earnings. The most serious complication for survivors of consequential CO exposure is delayed neurological or neurocognitive sequela which occurs in up to 50% of patients having symptomatic CO poisoning [3, 4].

The mechanism for CO poisoning includes lipid peroxidation, decreased oxygen carrying capacity, and inhibition of cytochrome *c* oxidase or Complex IV (CIV) [5–8]. The standard treatment for CO poisoning recommended by the Undersea & Hyperbaric Medical Society is hyperbaric oxygen (HBO) therapy to decrease half-life but also to prevent lipid peroxidation. There is one clinical trial which was the only RCT to meet CONSORT criteria that did find efficacy of HBO [9]. Despite recommendations, the effectiveness of HBO is widely debated with a Cochrane review concluding that existing randomized trials do not establish that administration of HBO to patients with CO poisoning reduces the incidence of adverse neurologic outcomes [10]. There is a clear need for a better understanding of the interaction of HBO and mitochondrial bioenergetic function in the face of CO poisoning.

In a previous study, we explored the logistics of measuring mitochondrial respiration that fits in an analytical timeframe useful to identify clinical abnormalities and we also found that clinically significant CO poisoning resulted in a decrease in key parameters of mitochondrial respiration regardless of the carboxyhemoglobin (COHb) level [8]. In this study, we used freshly obtained blood cells to perform simultaneous measurement of mitochondrial respiration and reactive oxygen species (ROS) production (e.g., hydrogen peroxide or H_2_O_2_ production). We evaluated blood samples from control subjects as well as subjects with clinically significant CO poisoning upon hospital presentation and also immediately after an initial treatment of HBO. The primary objective of this study was to assess changes in mitochondrial function that occur as a result of CO poisoning and subsequent HBO therapy.

## Methods

### Study design

**Fig. 1:**
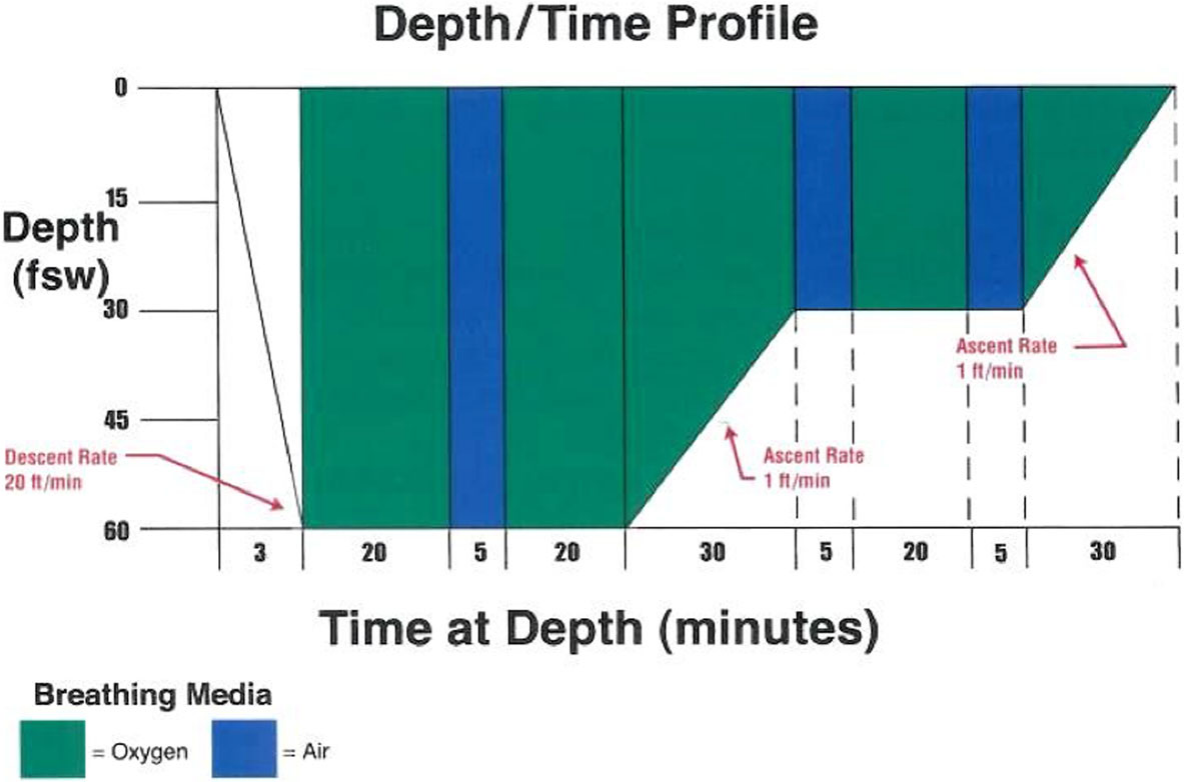
Treatment Table 5 for CO poisoning. The standard HBO protocol for patients with CO poisoning. All subjects in the CO group underwent HBO treatment at 2.8 ATA (60 ft of sea water pressure or 2.8 times of atmospheric pressure) for 120 min

The University of Pennsylvania Institutional Review Board approved this study and informed consent was obtained from all subjects. This was a two-group controlled experiment of acutely CO-poisoned subjects undergoing HBO treatment and control subjects. Whole blood was obtained from consented participants 1 h after emergency department (ED) presentation and immediately after undergoing HBO treatment. The first HBO treatment was at 2.8 ATA or atmospheres absolute pressure (60 ft of seawater pressure or 2.8 times of atmospheric pressure) for 120 min as seen in Fig. 1. We also obtained the following clinical information: history, physical exam, standard laboratory values, imaging studies, COHb, and hospital outcomes. Exclusion criteria for both groups include known malignancy, pregnancy, and oncology history with active chemotherapy by chart review and/or clinical assessment.

### Human blood cell preparation

All eligible subjects with confirmed CO poisoning underwent phlebotomy as part of standard care within 1 h of ED presentation and within 1 h after the initial HBO treatment. Samples were taken from the collection tubing at the same time as a planned blood phlebotomy. A volume of 15 ml was drawn in K_2_EDTA tubes to prohibit platelet activation. The following protocol was carried out to obtain a population of peripheral blood mononuclear cells (PBMCs): 15 ml of Ficoll-Paque™ PLUS was placed into a 50-ml Leucosep tube (Greiner Bio-one) which was centrifuged at 1000*g* for 1 min. During this process, 15 ml of the patient’s blood was added to a 50-ml Falcon tube followed by an addition of 15 ml of DPBS for a total of 30 ml in a 1:1 mixture. The 50-ml Falcon tube was gently inverted. Once the Leucosep centrifugation was completed, the 30 ml of blood was carefully pipetted and layered above the disc within the Leucosep tube. The Leucosep tube was centrifuged at 1000*g* for 10 min at room temperature after which approximately 4 ml of the buffy coat containing PBMCs was collected. This buffy coat sample was placed in a 15-ml tube followed by the addition of 4 ml of DPBS for a 1:1 mixture that was then centrifuged at 1000*g* for 7 min. The supernatant was discarded and the PBMC pellet was gently re-suspended in MiR05 medium for high-resolution respirometry. A cell count with trypan blue was performed with the Cell Countess II (Invitrogen) and cell viability was also assessed. To demonstrate that our sample preparation methodology yields PBMCs to a high degree, we performed flow cytometry analysis. Our use of the Leucosep tube resulted in samples having over 95% PBMCs and less than 5% erythrocytes and platelets combined.

### High-resolution respirometry

Respiration was measured at a constant temperature of 37 °C in a high-resolution oxygraph (Oxygraph-2k, OROBOROS Instruments) in 2-ml glass chambers with stirrer speed 750 rpm. Data were recorded with DatLab software 6 (OROBOROS Instruments) with the sampling rate set to 2 s. All experiments were performed at an oxygen concentration in the range of 210–50 μM O_2_ corrected for background flux. For respiration measurements in permeabilized cells, PBMCs were suspended in a mitochondrial respiration medium (MiR05) containing sucrose 110 mM, HEPES 20 mM, taurine 20 mM, K-lactobionate 60 mM, MgCl_2_ 3 mM, KH_2_PO_4_ 10 mM, EGTA 0.5 mM, and BSA 1 g/l and with pH 7.1 which allows for the simultaneous measurement of respiration and H_2_O_2_ production.

### Experimental protocol for reference protocol for permeabilized cells

**Fig. 2:**
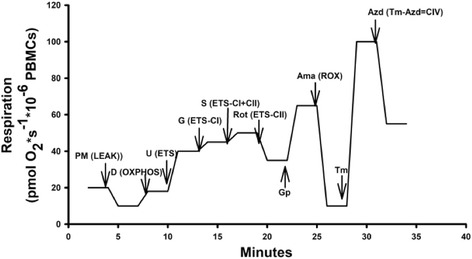
Reference protocol. The figure represents a standard tracing to obtain various coupling states in permeabilized cells. This reference protocol provides a common reference for comparison of respiratory control in a large variety of tissues and cell types. Each arrow represents a point of an injection with the corresponding compound and respiratory state in parentheses

We performed a specialized protocol using PBMCs to evaluate specific complex (CI-CIV) activity that requires controlled permeabilization with digitonin. This substrate-uncoupler-inhibitor titration (SUIT) protocol is referred to as reference protocol (RP) designed to provide common reference for comparison of respiratory control in a wide range of preparations ranging from isolated mitochondria, tissues, and various cell types including human blood cells utilized in this experiment [8, 11]. In order to access the electron transport system with saturating exogenous substrates and inhibitors, the plasma membrane was permeabilized with the detergent digitonin. A set of experiments were performed to establish the optimal concentration of digitonin to induce maximal permeabilization of the plasma membrane without affecting the outer or inner mitochondrial membrane. Figure 2 illustrates a typical respiration tracing utilized for our study with the following sequence of injections given in the RP to establish the respiratory capacities with electron flow through both CI and CII separately as well as convergent electron input via the Q-junction (CI + II). Both malate and pyruvate (PM) were added prior to the addition of cells. Once cells were added, routine respiration was allowed to be reached followed by titration with digitonin for permeabilization of the plasma membrane in the concentration described above which provides CI-linked LEAK state. Oxidative phosphorylation (OXPHOS) capacity of CI, driven by nicotinamide adenine dinucleotide (NADH)-related substrates, was evaluated by adding adenosine diphosphate-(D). Carbonyl cyanide m-chloro phenyl hydrazine (U) was titrated to obtain the maximal respiratory capacity or ETS. ETS is the maximum stimulation of mitochondrial respiration that is measured after the use of a protonophore that uncouples oxidative phosphorylation. ETS is often thought of as the maximal reserve for the cell to perform biochemical work. After U titration, glutamate (G) was added to obtain uncoupled CI respiration (ETS_CI_) that was added followed by succinate (S) to give uncoupled CI and CII respiration (ETS_CI + CII_). CI was inhibited by rotenone (Rot) to assess the ETS capacity supported by succinate through CII only or uncoupled CII respiration (ETS_CII_). After, glycerol 3-phosphate (Gp) was given to evaluate mitochondrial glycerophosphate dehydrogenase complex that feeds electrons directly to ubiquinone. Finally, electron flow through the ETS was inhibited by addition of antimycin-A (Ama), inhibiting CIII, providing the residual oxygen consumption (ROX) not related to non-mitochondrial oxygen consumption. ROX was subtracted from the different respiratory parameters in further analyses. CIV activity was then assessed with the addition of both ascorbate and N,N,N′,N′-tetramethyl-p-phenylenediamine dihydrochloride (Tm) followed by sodium azide (Azd). For correction of non-CIV-related oxygen consumption, the remaining chemical background was subtracted.

### Experimental protocol for the simultaneous measurement of hydrogen peroxide in permeabilized cell

In addition to the measurement of mitochondrial respiration in permeabilized cells, we also simultaneously measured the production of ROS as H_2_O_2_. All experiments were conducted with an O2k-Fluorometer LED2-Module connected to the O2k-Core using two fluorescence sensors green with the gain set to 1000 with a polarization of 400 mV (400 mV was an ideal balance between sensitivity and photobleaching). The following injections were performed prior to the injection of cells: (1) Amplex Red (AmR) was added followed by the addition of horseradish peroxidase that is necessary for the conversion of H_2_O_2_ and AmR to resorufin (fluorescence). Next superoxide dismutase was added to convert any superoxide to H_2_O_2_. The final injection prior to the addition of cells was freshly prepared H_2_O_2_ to test the AmR assay system and to allow calibration of the O2k-Fluorometer for AmR. Additionally, we have performed experiments with the use of resorufin as an internal control in a dose-dependent fashion to test for H_2_O_2_ signal and our prior work describes this technique [12, 13].

### Data analysis

Statistics were calculated by using Graph Pad Prism v.7 (GraphPad Software Inc.). Data were tested for normal distribution with the D’Agostino and Pearson omnibus normality test. Data are presented as mean ± SEM if not indicated otherwise. Differences between the control group and the CO group (before and after HBO therapy) were analyzed with repeated measures ANOVA and, when significant, paired Student’s *t* tests were performed between the respiratory states. The results were adjusted with Bonferroni correction for multiple tests. A *P* value of < 0.05 was considered statistically significant.

## Results

We enrolled a total of 17 subjects, including seven subjects with confirmed CO poisoning (CO group) and 10 healthy subjects (control group). The patient characteristics are presented in Table 1. The subjects in the control group were enrolled as a healthy convenience sample to serve as a comparison group for the measurement of mitochondrial respiration and ROS. The subjects in the control group were 50% male with a median age of 40 years (IQR, 36 to 51 years). Subject clinical characteristics included the following: six (60%) had no medical problems, one had obstructive lung disease, and three had hypertension. All subjects were discharged from the emergency department with no return visit on a 30-day chart review. The subjects in the CO group included five men (71%) and two women (29%). The median age of CO subjects enrolled was 67 (IQR, 47 to 68 years). Patient clinical characteristics included the following: four (57.1%) had no medical problems, one had Parkinson’s disease, and two had hypertension. Regarding the source of CO exposure, four (57.1%) were related to a faulty heat generator, two (28.6%) were related to occupational exposure involving machinery, and one (14.3%) was related to a car exhaust.

**Table 1:** Characteristics of subjects in the CO and control group

Characteristics	Carbon monoxide group (*n* = 7)	Control group (*n* = 10)
Age	67 (47–68)	40 (36–51)
Sex (%)		
Male	71	50
Female	29	50
Laboratory value		
COHb (%)	29 (28–32)	
Lactate (mmol/L)	2.78 (1.1–4.5)	
Clinical symptoms (%)		
Neurological		
Headache	57	
Seizure	14	
Syncope	43	
Cardiac		
Chest pain	14	
EKG changes	14	
Gastrointestinal		
Nausea and emesis	14	

Data presented as median (IQR) or as percentage

In the CO group, on arrival at the ED, four (57.1%) experienced syncope, one (14.3%) had a seizure, two (28.6%) presented with chest pain/EKG changes, and one (14.3%) presented with nausea and vomiting. Following arrival at the ED, six (85.7%) subjects were placed on non-rebreather mask and one (14.2%) was intubated for altered mental status. As an outcome index, one subject was admitted to the ICU, one was admitted to a general medicine floor for residual weakness, and five were discharged from the observation unit after undergoing three HBO dives. All seven CO subjects survived hospitalization.

**Fig. 3:**
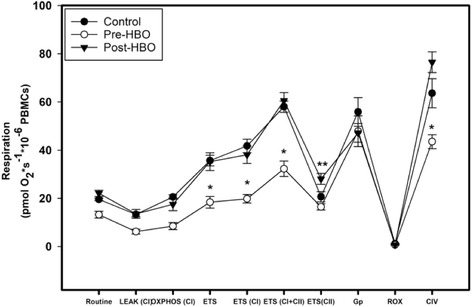
Mitochondrial respiration in PBMCs. Cellular mitochondrial respiration obtained in permeabilized PBMCs for the control group as well as the CO group (pre- and post-HBO). Values presented as mean ± SEM. *The CO group (pre-HBO) significantly different after their HBO treatment (post-HBO) and the control group (*P* < 0.001 for both). **The control group significantly different from the CO group both before and after HBO treatment (*P* < 0.0001 for both groups)

We compared mitochondrial function in PBMCs obtained from subjects in the CO group at two discrete time points, prior to HBO therapy and within 1 h after undergoing the initial HBO treatment as seen in Fig. 3. We also compared respiration results in the CO group (pre- and post-HBO) to the control group. All the units of respiration were measured in pmol O_2_ × s^− 1^ × 10^− 6^ PBMCs. Table 2 contains both mitochondrial respiration and H_2_O_2_ production at all the key respiratory states.

**Table 2:** Mitochondrial respiration and H_2_O_2_ production

	Control group		CO group (pre-HBO)		CO group (post-HBO)	
Respiratory states	Mean	SEM	Mean	SEM	Mean	SEM
Mitochondrial respiration (measured in pmol O_2_ × s^− 1^ × 10^− 6^ PBMCs)						
Routine	19.56	0.54	13.23	1.42	22.10	1.28
LEAK	13.33	0.97	6.19	0.99	13.54	1.81
OXPHOS	20.57	0.87	8.47	1.45	17.51	2.58
ETS	35.66	2.20	18.39	2.42	35.23	3.70
ETS-CI	41.73	2.76	19.81	1.77	38.07	3.61
ETS-CI&II	58.04	2.33	32.27	3.17	60.54	3.32
ETS-CII	20.67	2.11	16.44	1.30	28.07	2.25
Gp	55.86	5.96	47.86	6.36	47.15	3.88
ROX	1.17	0.20	0.73	0.19	0.76	0.20
CIV	63.60	6.03	43.52	2.92	76.50	4.30
Mitochondrial H_2_O_2_ production (measured in pmol H_2_O_2_ × s^−1^ × 10^−6^ PBMCs)						
Routine	0.06	0.02	0.04	0.02	0.16	0.07
LEAK	0.18	0.05	0.24	0.13	0.92	0.14
OXPHOS	0.25	0.05	0.16	0.05	0.93	0.22
ETS	0.22	0.04	0.14	0.02	0.85	0.20
ETS-CI	0.22	0.04	0.13	0.02	0.66	0.21
ETS-CI&II	0.22	0.04	0.17	0.02	1.14	0.32
ETS-CII	0.26	0.06	0.23	0.04	0.71	0.08
ROX	0.57	0.15	0.31	0.06	1.59	0.42

Cellular mitochondrial respiration and hydrogen peroxide production obtained in permeabilized PBMCs between the control group, CO (pre-HBO) group, and the same CO group after undergoing HBO treatment (post-HBO). Values presented as mean ± SEM

The following key parameters of respiration was significantly lower in the CO group (pre-HBO) when compared to the control group, respectively: OXPHOS_CI_, ETS or maximal respiration, ETS_CI_, ETS_CI + CII_, and CIV respiration. There were no differences in R, LEAK, ETS_CII_, Gp, and ROX between the two groups. There was a significant overall increase in respiration in the CO group after they underwent HBO when compared to pre-HBO treatment except with no differences in respiration with R, LEAK, Gp, and ROX. Also of note, the control group and the CO group (post HBO) were similar in all respiratory states.

**Fig. 4:**
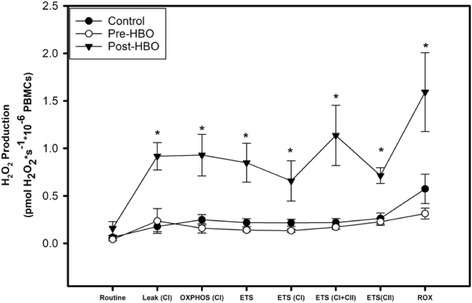
ROS production in PBMCs. Measurement of ROS production using the Amplex Red assay. H_2_O_2_ levels are measured in the presence of superoxide dismutase to ensure that all superoxide is converted into H_2_O_2_. Values presented as mean ± SEM. *The CO group (pre-HBO) is significantly different after their HBO treatment (post-HBO) and from the control group (*P <* 0.001 for both groups)

We also determined the rate of H_2_O_2_ production (as well as superoxide converted to H_2_O_2_ with the administration of SOD) simultaneously with the measurement of respiration in Fig. 4. The final H_2_O_2_ production values were obtained after correction for both background fluorescence and changes in sensitivity over time. There were no differences in H_2_O_2_ production between the CO group (pre-HBO) versus the control group. There was a significant increase in all key parameters of H_2_O_2_ production measured in pmol H_2_O_2_ × s^− 1^ × 10^− 6^ PBMCs in the CO group after they underwent HBO treatment when compared to pre-HBO treatment with the exception of routine indicating an effect of HBO therapy on H_2_O_2_ production. H_2_O_2_ production was also significantly higher in the same CO group after HBO treatment when compared to the control group with the exception of R.

## Discussion

The present study investigates the changes in both mitochondrial respiration and hydrogen peroxide production in PBMCs obtained from subjects who were poisoned by CO undergoing their first treatment of HBO (2.8 ATA for 120 min). The findings of this study were that PBMCs obtained from subjects with CO poisoning when compared to the control group exhibited an overall decrease in mitochondrial respiration. Importantly, for the CO subjects who underwent the first treatment of HBO, there was an overall increase in mitochondrial respiration post HBO with the exception of glycerophosphate dehydrogenase complex (CGpDH) activity which is a complex of the electron transfer system localized at the outer face of the inner mt-membrane. In regard to H_2_O_2_ production, there was an overall increase in H_2_O_2_ production along the main parameters of mitochondrial respiration post HBO treatment for the CO group.

One of findings in this study was a decrease in CIV respiration in the CO group when compared to the control group. We previously showed a decrease in CIV respiration in PBMCs obtained from a subset of subjects with severe CO poisoning related to death. In addition to the effect of CO on CIV activity, we also found that the measurement of mitochondrial respiration served as a better clinical indicator of outcome when compared to a COHb level [8]. Earlier studies have also demonstrated CIV inhibition where one study showed sustained inhibition [14–16]. However, there are a number of methodological issues with these early studies to consider. The one study that demonstrated sustained CIV inhibition only contained three subjects who were also actively smoking during the repeat measures of CIV activity [14]. It is known that cigarettes contain both CO and a small amount of cyanide, which is a significant cofounding factor. Another limitation of another study include the use of an in vitro model using striated muscle artificially exposed to CO and frozen samples with delayed results unlikely to affect clinical care utilizing the use of spectrophotometry [15, 16]. In general, spectrophotometry requires homogenization of samples with a high risk of mitochondrial damage and more artificial conditions when compared to HRR.

One of the strengths of performing HRR in permeabilized cells is that it allows examination of a variety of respiratory states at each complex. In this current study, there was a decrease in CIV respiration most likely secondary to inhibition from CO. This RP SUIT provides various coupling states, and in particular, CIV respiration can be obtained after the inhibition of CIII with Ama. CIV is provided with specific compounds as described in our method to obtain specific CIV respiration. This specific sequence of injections ensures that the CIV respiration obtained is not from the contribution of other complexes. In another study we performed, intact PBMCs obtained from healthy subjects exposed to cyanide (CIV inhibitor) resulted in an overall decrease in mitochondrial respiration in all the main parameters of respiration measured in intact cells that include R and ETS [17]. In our present study, the overall improvement in respiration in subjects who were treated with HBO therapy may have been due to the enhanced removal of CO at CIV restoring electron flow upstream. While based on the current literature, inhibition of CIV resulting from CO binding effect will slow down respiration at other complex that is the likely explanation for our findings. Also another important consideration is that our CO group had variable clinical severity of CO poisoning which can also account for this finding. A third consideration is that CO also causes hypoxia where CI is sensitive to hypoxia so the decrease in CI activity is more likely from CI injury as opposed to direct CI inhibition [18, 19].

We utilized an innovative technique of simultaneously measuring both cellular respiration and H_2_O_2_ production with the AmR system. This method has been applied in a limited number of studies but provides important information regarding H_2_O_2_ production in relation to cellular respiration [13, 20]. The measurement of ROS in real time can be technically challenging as it depends on a number of factors such as the type of ROS, timing, and technique. For example, the measurement of superoxide is technically more challenging, as this species is short-lived and immediately converted to H_2_O_2_ through SOD [21]. The measurement of H_2_O_2_ production confers certain advantage as H_2_O_2_ is a more stable compound and its measurement is well established. In our study, we did use SOD that converts all superoxide radicals to H_2_O_2_ so the H_2_O_2_ production we obtained reflects both superoxide and H_2_O_2_ production.

In addition to measuring mitochondrial respiration, we also simultaneously measured H_2_O_2_ production in PBMCs obtained from subjects poisoned with CO compared to the control group. We found that there was no difference in H_2_O_2_ production between the CO (pre-HBO) group and the control group. This is consistent as CI and CIII are considered the major sites for ROS production whereas CIV is generally not a source of ROS production as seen with our results. However, there is some evidence that CIV inhibition can lead to increased reduction of redox centers in CI or CII with potential ROS generation [22, 23]. The results in our study do support that CIV inhibition with CO alone does not result in significant ROS production. It is important to note that the use of strongly redox active substances such as cytochrome *c* are incompatible with the AmR assay so once CIV substrates were given to obtain CIV respiration only H_2_O_2_ production up until the injection of Ama is a reliable reflection of H_2_O_2_ production.

All of the CO subjects underwent HBO therapy, and repeat measurements in PBMCs obtained from the CO group immediately following HBO showed significant increased H_2_O_2_ production in the majority of the parameters of respiration. HBO therapy is the established treatment modality for CO poisoning. Subjects who undergo HBO breathe 100% O_2_ while exposed to increased atmospheric pressure. Exposure to greater than 1 atmosphere of oxygen results in an increase in the production of ROS, and this increased production of ROS is utilized clinically for other indications of HBO such as wound healing. ROS as well as reactive nitrogen species includes a variety of species such as superoxide radicals and H_2_O_2_ that are predominantly generated by the mitochondria in addition to other organelles such as the endoplasmic reticulum and peroxisomes [24]. ROS play a critical role in the function of the mitochondria, involved in signaling, and participate in important redox reactions [25]. Despite the multitude of biological functions ROS participate in, excessive generation of ROS has been implicated in a number of pathological disease states such as diabetes and heart disease but include acute illnesses such as sepsis and cardiac arrest [26–28].

Our results show that the initial HBO treatment in the CO group resulted in a significant increase in H_2_O_2_ production most likely related to HBO with increased oxygen. It should be noted that H_2_O_2_ production is elevated in virtually all states in the CO group after HBO treatment, long before addition of Ama so it is not the use of Ama that results in increased H_2_O_2_ as Ama is used in both groups. Equation 1 illustrates the time-dependent relationship between oxygen concentration and superoxide formation:

1 $$ \frac{d\left[{\mathrm{O}}_2\right]}{dt}=k\ \left[{\mathrm{O}}_2\right]\left[R\bullet \right] $$

The overall production of superoxide will vary depending on tissue type, the respiratory state of the mitochondria, and also mitochondrial inhibition. In general, CI and CIII are the primary sites of ROS generation which may be further increased with the use of certain inhibitors such as Rot for CI and Ama for CIII although results have varied depending on the tissue or cell type [22].

In our study, when comparing CIII (Ama) with CI (Rot) inhibition, there was no significant difference (*P* = 0.06). Another factor to consider for the elevated H_2_O_2_ in our study is the use of immune cells to measure H_2_O_2_. Certain immune cells such as neutrophils mediate elimination of foreign organisms such as bacteria and fungi with superoxide that is produced by NADPH oxidases located on the cell membrane [29]. Another source of ROS may be from immune cell activation. However, it has been shown that HBO results in decreased immune response from specific cell types such as monocytes which is part of the reason HBO is used for wound healing [30]. The implications of increased H_2_O_2_ production in the setting of CO poisoning and treatment is an important question as it is not clear if this is pathological or due to increased cellular messaging. Our study does not address the fate of the H_2_O_2_ produced as it may go on to become H_2_O or a hydroxyl radical, the latter being a very potent oxidant. This would warrant further study with HBO as this may help to explain the mixed results of the effectiveness of HBO in the treatment of CO poisoning.

The primary objective of this study was to examine measurement of both respiration and H_2_O_2_ production, as these may have important roles in prognosis and treatment as a clinical bedside tool. In our study, we were able to obtain both respiration and H_2_O_2_ production in the CO groups prior to the subjects undergoing their initial HBO treatment. Specifically, we are able to obtain results from the time of blood sample drawn to data in under 2 h [31]. It is not uncommon that the time required for patients to undergo initial evaluation for CO poisoning in the ED and then be transported to facilities for HBO treatment is greater than this. Our work describes the measurement of both mitochondrial respiration and H_2_O_2_ production in CO poisoning. It has potential application as a bedside tool in the acute care and treatment of patients having other forms of metabolic dysfunction resulting from poisoning or sepsis [31].

### Limitations

There are some limitations to consider with the present study. An important limitation is the lack of inclusion of a control group of healthy subjects who undergo HBO without having any clinical need for such therapy. HBO therapy, while having a favorable safety profile, is not without complications. The risk of barotrauma increases even at 1.2 ATA and subjects may experience otalgia and barotrauma to other areas such as the sinuses. Another complication is oxygen-induced seizure with a risk of up to 1/100 at 2.8 ATA [32]. In consideration of these complications, we chose not to incorporate volunteer subjects exposed to HBO therapy. Future studies could perform testing on patients not poisoned with CO who receive hyperbaric therapy for a non-acute illness such as wound healing although the depth of the dive may differ.

Another limitation is the lack of CO-exposed subjects who did not undergo HBO therapy. It is not clear how both mitochondrial respiration and H_2_O_2_ production would have changed without HBO treatment. For ethical reasons, HBO therapy was not withheld from any CO subject who met indications for HBO treatment. However, we note that repeat measures of mitochondrial function could be performed on patients who did not undergo HBO, as many patients with CO poisoning do not receive HBO [33]. A third limitation of this study is the connection between the use of human blood cells as a marker of mitochondrial function in vital organs such as the brain and heart that are affected in CO poisoning. An animal model of hemorrhagic shock has been used to evaluate blood cells as a marker of organ mitochondrial function [34]. The advantage of using blood cells as a surrogate biomarker to assess organ mitochondrial function is very attractive for both clinical and research reasons. Blood cells are easily accessible with minimal risk via venipuncture, and samples can be obtained repeatedly to assess mitochondrial function over time and in response to treatment. Finally, another consideration is the novelty of simultaneous measurement of respiration and H_2_O_2_ production. At this time, the current ROS that can be readily tested for is H_2_O_2_ and superoxide to some extent. A limitation is that other ROS such as hydroxyl radical or other nitrogen-based species are not tested so it may give an incomplete picture involving ROS.

## Conclusions

In this study**,** PBMCs obtained from subjects with confirmed CO poisoning have an overall decrease in respiration but similar H_2_O_2_ production when compared to controls. In the CO group who all underwent treatment with HBO, PBMCs obtained immediately after the first treatment of HBO displayed an overall increase in respiration and H_2_O_2_ production. The study findings demonstrate that treatment with HBO resulted in improved cellular respiration but higher H_2_O_2_ production. It is unclear if the increased production of H_2_O_2_ in HBO treatment is detrimental.

## Abbreviations

Ama: Antimycin—Complex III inhibitor
AmR: Amplex red—extrinsic fluorophore for measurement of hydrogen peroxide production as resorufin
Azd: Azide—Complex IV inhibitor. To obtain CIV respiration, once Tm is given, this is followed by Azd. The difference between the two will provide CIV respiration
CI-IV: Complex I-IV
D: Adenosine diphosphate (ADP)—substrate of ANT and ATP synthase in the phosphorylation system
G: Glutamate—supporting an N-linked (Complex I) pathway control state
Gp: Glycerophosphate—substrate for electrons donation to ubiquinone
HRR: High-resolution respirometry (Measured with the OROROROS O2K)
H_2_O_2_: Hydrogen peroxide—one of several reactive oxygen intermediates
L: LEAK respiration—dissipative component of respiration which is not available for performing biochemical work
P: OXPHOS capacity—is the respiratory capacity of mitochondria in the ADP-activated state of oxidative phosphorylation
PM: Pyruvate and malate—used to obtain LEAK state
R: ROUTINE respiration—cellular energy demand, energy turnover and the degree of coupling to phosphorylation often representing steady state
Rot: Rotenone—Complex I inhibitor
ROX: Residual oxygen consumption—respiration due to oxidative side reactions remaining after inhibition of the electron transport system (Rot and Ama)
RP: Reference protocol—provides a common baseline for comparison of mitochondrial respiratory control in a large variety of species, tissues and cell types (see Fig. 2 for example of typical tracing)
S: Succinate—Complex II substrate
SUIT: Substrate-uncoupler-inhibitor titration—sequence of compounds given to obtain various coupling states
Tm: Denotes the injection of N,N,N′,N′-tetramethyl-p-phenylenediamine dihydrochloride, which is an artificial substrate for reducing cytochrome *c* in the respirometric assay for cytochrome *c* oxidase (CIV) activity followed by ascorbate that maintains N,N,N′,N′-tetramethyl-p-phenylenediamine dihydrochloride in a reduced state
U: Carbonyl cyanide m-chlorophenyl hydrazine—a protonophore (H+ ionophore) and is used as a potent chemical uncoupler of oxidative phosphorylation
